# Artificial intelligence and machine learning in orthopedic surgery: a systematic review protocol

**DOI:** 10.1186/s13018-020-02002-z

**Published:** 2020-10-19

**Authors:** Nicola Maffulli, Hugo C. Rodriguez, Ian W. Stone, Andrew Nam, Albert Song, Manu Gupta, Rebecca Alvarado, David Ramon, Ashim Gupta

**Affiliations:** 1 Department of Musculoskeletal Disorders, School of Medicine and Surgery, University of Salerno, Fisciano, Italy; 2 San Giovanni di Dio e Ruggi D’Aragona Hospital “Clinica Orthopedica” Department, Hospital of Salerno, Salerno, Italy; 3 Queen Mary University of London, Barts and the London School of Medicine and Dentistry, Centre for Sports and Exercise Medicine, London, England; 4 School of Osteopathic Medicine, University of the Incarnate Word, San Antonio, TX USA; 5 Future Biologics LLC, 1110 Ballpark Ln Apt 5109, Lawrenceville, GA 30043 USA; 6 South Texas Orthopaedic Research Institute, Laredo, TX USA; 7 Texas A&M International University, Laredo, TX USA; 8 BioIntegrate, Lawrenceville, GA USA; 9 Veterans in Pain, Los Angeles, CA USA

**Keywords:** Machine learning, Artificial intelligence, Orthopedic surgery, PRISMA

## Abstract

**Background:**

Artificial intelligence (AI) and machine learning (ML) are interwoven into our everyday lives and have grown enormously in some major fields in medicine including cardiology and radiology. While these specialties have quickly embraced AI and ML, orthopedic surgery has been slower to do so. Fortunately, there has been a recent surge in new research emphasizing the need for a systematic review. The primary objective of this systematic review will be to provide an update on the advances of AI and ML in the field of orthopedic surgery. The secondary objectives will be to evaluate the applications of AI and ML in providing a clinical diagnosis and predicting post-operative outcomes and complications in orthopedic surgery.

**Methods:**

A systematic search will be conducted in PubMed, ScienceDirect, and Google Scholar databases for articles written in English, Italian, French, Spanish, and Portuguese language articles published up to September 2020. References will be screened and assessed for eligibility by at least two independent reviewers as per PRISMA guidelines. Studies must apply to orthopedic interventions and acute and chronic orthopedic musculoskeletal injuries to be considered eligible. Studies will be excluded if they are animal studies and do not relate to orthopedic interventions or if no clinical data were produced. Gold standard processes and practices to obtain a clinical diagnosis and predict post-operative outcomes shall be compared with and without the use of ML algorithms. Any case reports and other primary studies assessing the prediction rate of post-operative outcomes or the ability to identify a diagnosis in orthopedic surgery will be included. Systematic reviews or literature reviews will be examined to identify further studies for inclusion, and the results of meta-analyses will not be included in the analysis.

**Discussion:**

Our findings will evaluate the advances of AI and ML in the field of orthopedic surgery. We expect to find a large quantity of uncontrolled studies and a smaller subset of articles describing actual applications and outcomes for clinical care. Cohort studies and large randomized control trial will likely be needed.

**Trial registration:**

The protocol will be registered on PROSPERO international prospective register of systematic reviews prior to commencement.

## Background

Artificial intelligence (AI) and machine learning (ML) are interwoven in our everyday lives: from the way our email inbox is organized to the algorithms that dictate our Netflix preferences. In comparison, the adoption of ML in the medical field has been relatively slower, but there has been a recent surge in growth. AI and ML have been applied in various fields of medicine such as mental health, cardiology, dermatology, and radiology, where they have seen the greatest use [1, 2]. AI and ML are gaining increasing interest given their success in these fields of medicine, where in some cases they are able to outperform human specialists [3]. The application of AI and ML in the orthopedic field is still in an earlier stage compared to other areas of medicine [1]. Structured research frameworks such as cohort studies and randomized controlled trials, as well as more experimental research, are still needed for AI and ML to be widely accepted in orthopedics [1].

ML utilizes computer algorithms and statistics to identify complex patterns and trends within the data that otherwise would not be distinguishable by humans [4]. ML can “learn” patterns from data and produce models linking covariates to a target variable of interest and build models to describe the behavior of a system [4, 5]. In the field of medicine, ML can compile data from imaging and laboratory tests, and electronic medical records to guide physicians in formulating more efficacious and productive decisions [1].

Two broad categories of ML are generally employed in medicine, depending on the task: supervised learning and unsupervised learning [5]. Supervised learning focuses on choosing among subgroups to describe a new instance of data and estimating an unknown parameter [5]. For example, an automated interpretation of an electrocardiogram where a specific pattern is linked to a set of diagnoses, or how a lung nodule from a chest radiograph is detected automatically [5]. In contrast, unsupervised learning centers on the patterns or groupings within the data rather than predicting an output [5]. The goal of unsupervised learning is to uncover the hidden structure in the data and learn its pattern [2]. For example, endomyocardial biopsies can be taken and histologically examined to identify cellular composition that can aid in developing targeted therapy for myocarditis [5]. The category of machine learning applied depends on the needs of the patient and the physician.

ML holds tremendous potential to improve the quality of life for patients in a plethora of medical specialties including mental health. The Kyoto Prefectural University of Medicine utilized simple linear regression and L1-sparse canonical correlation analysis ML algorithms to identify a clinical biomarker for obsessive-compulsive disorder (OCD) [6]. Using magnetic resonance imaging (MRI), the biomarker was able to distinguish between patients with OCD and non-affected human controls with 73% accuracy [6]. Repeating the exam with a different MRI machine and subset of patients led to 70% accuracy [6]. While only 108 participants took part in this study, the reproducibility of the data holds promise for future clinical applications. Furthermore, another study analyzed tens of thousands of Instagram photos to identify markers of depression [7]. Researchers utilized color analysis, Instagram metadata components, and algorithm face detection to produce predictive models for depression screening. These predictive models were able to outperform general practitioners in diagnosing depression [7]. Results held true even for patients who did not have an initial diagnosis of depression [7]. The simple utilization of such ML models in the primary care office could greatly increase the early and successful diagnosis of depression.

Radiology is another field which has quickly adopted ML. In some applications, ML performed as well or even better than orthopedic surgeons in fracture detection of the upper limb, ankle, and spine [8]. ML can also be integrated into current imaging systems making them “intelligent,” leading to faster imaging speeds and the ability to offer modifications to ongoing magnetic resonance imaging sequences to visualize a lesion more accurately [2]. This can also be done by integrating the use of information from a patient’s medical records, allowing the program to determine the most appropriate patient-specific imaging examination and protocol [8]. It even has the potential to automatically detect incidental findings on imaging and learn how to identify critical findings, such as a pneumothorax [2]. The use of ML does not aim at replacing the radiologist but augment their workflow and enhance their diagnostic accuracy [2]. These algorithms are able to identify findings that might not be so easily seen by the human eye, such as using the variations in intensity on MRI to predict O6-methylguanine methyltransferase gene promoter methylation in glioblastoma multiforme tumors [9].

Orthopedic surgery is one of the most technologically innovative fields in medicine. Nevertheless, AI and ML adoption is still in a preliminary phase in orthopedics [1]. ML can be used to provide a patient-specific predicted rate of post-operative complications, predict injury risk patterns, and guide clinical decision-making [8, 10]. Considering the recent growth of ML in the specialty and the quantity of new research being published, a systematic review is required. There are over 3300 published articles relating to AI and ML in orthopedics, with over 1100 of those having been published in the last 2 years alone. Given the marked increase in the number of peer-reviewed publications, the primary objective of this review is to provide an update on the advances of AI and ML in the field of orthopedic surgery. The secondary objectives of this review are to evaluate the applications of AI and ML in providing a clinical diagnosis and predicting post-operative outcomes and complications in orthopedic surgery.

## Methods

### Protocol and registration

The protocol will be registered on the PROSPERO international prospective register of systematic reviews. The systematic review will follow the Preferred Reporting Items for Systematic Reviews and Meta-Analyses (PRISMA) statement and guidelines [11, 12].

### Eligibility criteria

For the initial literature search, the PICOS (Population, Intervention, Comparison, Outcome, Study Design) framework will be utilized for the eligibility criteria [13]. The following characteristics for clinical studies are:

#### Population

Studies involving human models from adult participants (18 years or older) will be considered for review, without any geographical exclusion criteria. Studies meeting eligibility criteria will apply to orthopedic interventions and acute and chronic orthopedic musculoskeletal injuries. Articles will be excluded from eligibility if they are animal studies or do not relate to orthopedic intervention.

#### Intervention

The studies considered will present ML models employing deep learning as an intervention with the aim of providing diagnosis or clinical prognosis of an orthopedic surgery intervention. The intervention may be used by itself or with other methods. Studies will be excluded if no clinical data were used. Considering that there is no single best ML model, various models will be used.

#### Comparison

Gold standard processes and practices to obtain a clinical diagnosis and predict post-operative outcomes shall be compared with and without the use of ML algorithms.

#### Outcomes

The primary outcome is the evaluation of ML models and how accurately they can provide a clinical diagnosis and how accurately they can predict post-operative outcomes and complications of orthopedic surgery interventions.

#### Study design

Any case reports and other primary studies assessing the prediction rate of post-operative outcomes or the ability to identify a diagnosis in orthopedic surgery will be included. Systematic reviews or literature reviews will only be examined to identify further studies for inclusion, and the results of the meta-analysis will not be included in the analysis. Regarding publication year, all studies published to date will be included.

### Information sources

A systematic search will be conducted in PubMed, ScienceDirect, and Google Scholar databases of English, Italian, French, Spanish, and Portuguese language articles published before July 2020. Secondary searching of reference lists of key articles and reviews will be undertaken in order to identify any additional studies potentially missed in the electronic search.

### Search

The PRISMA checklist and flow diagram will be used as the eligibility and inclusion criteria during the search and selection process. A web-based reference software system (RefWorks) will be used for data management.

### Study selection

The study selection process will entail an initial review by two different reviewers of all titles and abstracts. These findings will be uploaded to the web-based reference software system. This will be followed by a second review, again by two independent reviewers, of the full-text articles. This will ensure that the remaining articles meet the inclusion criteria. Discrepancies at any level will be resolved through discussion with a third reviewer.

### Data collection

Two independent reviewers will perform data extraction from articles which will meet the inclusion criteria. The data extracted and synthesized will include study characteristics, application of the ML model, outcome measurement, outcome assessment, and complications or adverse events reported. Forms will be customized during the data extraction and collection process. Primary authors will be contacted via email if any information requires clarification.

### Data items

Relevant items related to study characteristics such as authors, study design, and year of publication will be extracted and included. The characteristics of the ML software and its specific use in the prediction of a diagnosis or treatment outcome will also be included. Outcome measures relating to predicting a diagnosis will include negative predictive value, positive predictive value, sensitivity, and specificity. Measures extracted and included for the prediction of post-operative outcomes will include function, general outcomes, complications, success, and survival.

### Risk of bias

Multiple resources will be used to assess the risk of biases and shall be reported as low risk, moderate risk, or high risk of biases. Ten domains will be addressed related to selection bias, performance bias, detection bias, attrition bias, reporting bias, etc. The Risk Of Bias In Non-randomized Studies of Interventions (ROBINS-I) tool assesses observational and quasi-randomized studies [14]. Seven domains will be used in assessing risk, including confounding, participant selection bias, classification bias, deviation bias, bias from missing data, outcome measurement bias, and bias in the selection of reported results. Studies will be categorized into no information or a low, moderate, serious, or critical risk of bias. For randomized control trials, the Risk of Bias 2 (RoB 2) tool will be used to establish the risk of bias [15]. Five domains of biases will be analyzed, including those from the randomization process, those arising from deviations from intended interventions, those from missing outcome data, those found in measuring the outcome, and those found in the selection of the reported result. All included studies will be independently scored by two reviewers, and a discussion will facilitate consensus of the biases risk levels.

### Data synthesis and meta-analysis

Guidelines published by Hooijmans et al. will be used for the data synthesis and meta-analysis [16]. A random-effects meta-analysis followed by subgroup analysis will be performed if appropriate, given the anticipated heterogeneity among studies. Sources of potential heterogeneity include the ML software used, what it was used for (diagnosis or treatment), and the treatment population and indication. The results of the meta-analysis will be summarized appropriately with emphasis on design and outcome measures.

## Discussion

Machine learning has incredible potential given its ability to process large amounts of patient information and predict patient outcomes, a reason why it is important for orthopedic surgeons to have the most current information. The proposed systematic review will evaluate the advances of AI and ML in the field of orthopedic surgery. Given the recent surge in published peer-reviewed articles in ML and AI in orthopedics, we expect to identify a large quantity of interesting and relevant yet uncontrolled studies, and a smaller subset of investigations describing actual applications and outcomes for clinical care. Research frameworks such as cohort studies and large randomized control trial will likely be needed.

### Documenting protocol amendments

Protocol amendments and updates will be documented via PROSPERO online register. The description of the changes will be recorded, dated, and accessible along with the most up-to-date version within the record audit trail under the protocol registration.

## Data Availability

The results of this review will be published in a relevant scientific journal or presented at national or international conferences (‘Publications’) by the investigators.

## Abbreviations

AI: Artificial intelligence
ML: Machine learning
MRI: Magnetic resonance imaging
OCD: Obsessive-compulsive disorder
PICOS: Population, Intervention, Comparison and Outcome
PRISMA: Preferred Reporting Items for Systematic Reviews and Meta-Analyses
RoB 2: Risk of Bias 2
ROBINS-I: Risk OF Bias in Non-randomized Studies of Interventions
